# Classification-related approach in the surgical treatment of thoracolumbar fractures

**DOI:** 10.4103/0019-5413.36996

**Published:** 2007

**Authors:** R Lukas, J Sram

**Affiliations:** Trauma centre with Spinal unit, Liberec Regional Hospital, Czech Republic

**Keywords:** Fractures of thoracolumbar spine, fracture classification, surgical approach

## Abstract

**Background:**

Advanced diagnostic tools, classification systems and accordingly selected surgical approaches are essential requirements for the prevention of failure of surgical treatment of thoracolumbar fractures. The present study is designed to evaluate the contribution of classification to the choice of a surgical approach using the current fracture classification systems.

**Materials and Methods:**

We studied prospectively a group of 64 patients (22 females, 42 males) of an average age of 43 years, all operated on for thoracolumbar fractures during the year 2001. The AO-ASIF classification was used preoperatively with all imaging studies (X-ray, computed tomography (CT) and magnetic resonance imaging (MRI)). When the damage was detected only in the anterior column (A type), an isolated anterior stabilization (n = 22) was preferred. If the MRI study disclosed an injury in the posterior column, a posterior approach (n = 20) using the internal fixator was chosen. Injuries involving the posterior column (B or C type) were classified additionally according to the load-sharing classification (LSC). If LSC gave six or more points, treatment was completed with an anterior fusion. The combined postero-anterior procedure was carried out 22 times. The minimum followup period was 22 months.

**Results:**

Neither implant failure and nor significant loss of correction were observed in patients treated with anterior or combined procedures. The average loss of correction (increase of kyphosis) in simple posterior stabilization was 3.1 degree.

**Conclusion:**

Complex fracture classification helps in the selection of the surgical approach and helps to decrease the chances of treatment failure.

Inspite of progress in imaging, understanding of spinal stability^1^ and modern classification systems, there is no generally accepted consensus regarding the type of the surgical approach in the treatment of thoracolumbar fractures. Verlaan *et al*. showed that a long posterior stabilization is the most frequently used treatment modality.^2^ Few spine surgeons prefer the anterior approach.^3^–^5^ Surgeries irrespective of the fracture pattern and residual stability, are associated with remarkable loss of correction.^6^–^10^

The ligamentous disruption is best evaluated by magnetic resonance imaging (MRI).^11^ The AO-ASIF classification of thoracolumbar fractures describes a fracture by the types and number of damage in the anterior and posterior columns.^12^ The load sharing classification (LSC)^9^ evaluates the damage in the anterior column. Both these classification systems—AO-ASIF and LSC offer very complex characterization of the individual fracture pattern and may be used as guidance for the decision-making regarding the surgical approach. By adjusting the surgical plan to the fracture pattern, the results of treatment might be improved.^6^ This study is designed to evaluate the contribution of the classification-related issues to the surgical approach using the current fracture classification systems.

## MATERIALS AND METHODS

We prospectively studied a series of 64 patients with unstable fractures of the thoracolumbar spine who had been treated surgically in 2001. Patients with multiple fractures, osteoporosis and spinal cord injury were excluded from the study. The series consisted of 22 women and 42 men with a mean age of 43 years (19-71 yrs).

All patients were investigated preoperatively by plain X-ray, CT and MRI and their injury examined using the AO-ASIF classification. When the damage was detected only in the anterior column (AO-ASIF A type), an isolated anterior approach was preferred. Stabilization was carried out using an anterior angle-stable device (MACS-TL) and a spacer or tricortical bone graft. If an injury of the posterior column (bony or ligamentous) was disclosed, the posterior approach using the internal fixator was chosen. In these injuries (AO-ASIF B or C type), damage of the anterior column was classified additionally according to the load-sharing classification (LSC).^9^ If LSC gave more than six points, treatment was completed with an anterior fusion.

The patients were divided into three groups:

- group I (n = 22): “A” type fractures treated with simple anterior stabilization and fusion
- group II (n = 22): “B or C” type of fractures with LSC scoring equal or higher than 6 points treated using combined posteroanterior procedure [Figure 1a–d].

**Figure 1a:**
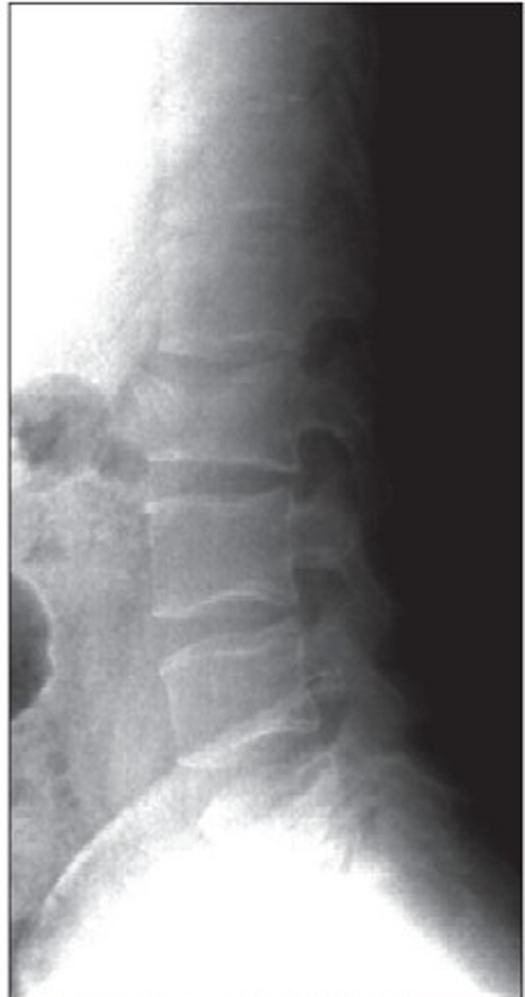
Preoperative X-ray of L1 fracture, B type with LSC 7

**Figure 1b:**
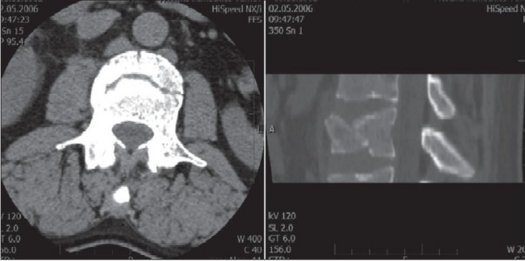
CT scan axial cut and sagittal reconstruction shows a pincer fracture of the vertebral body

**Figure 1c:**
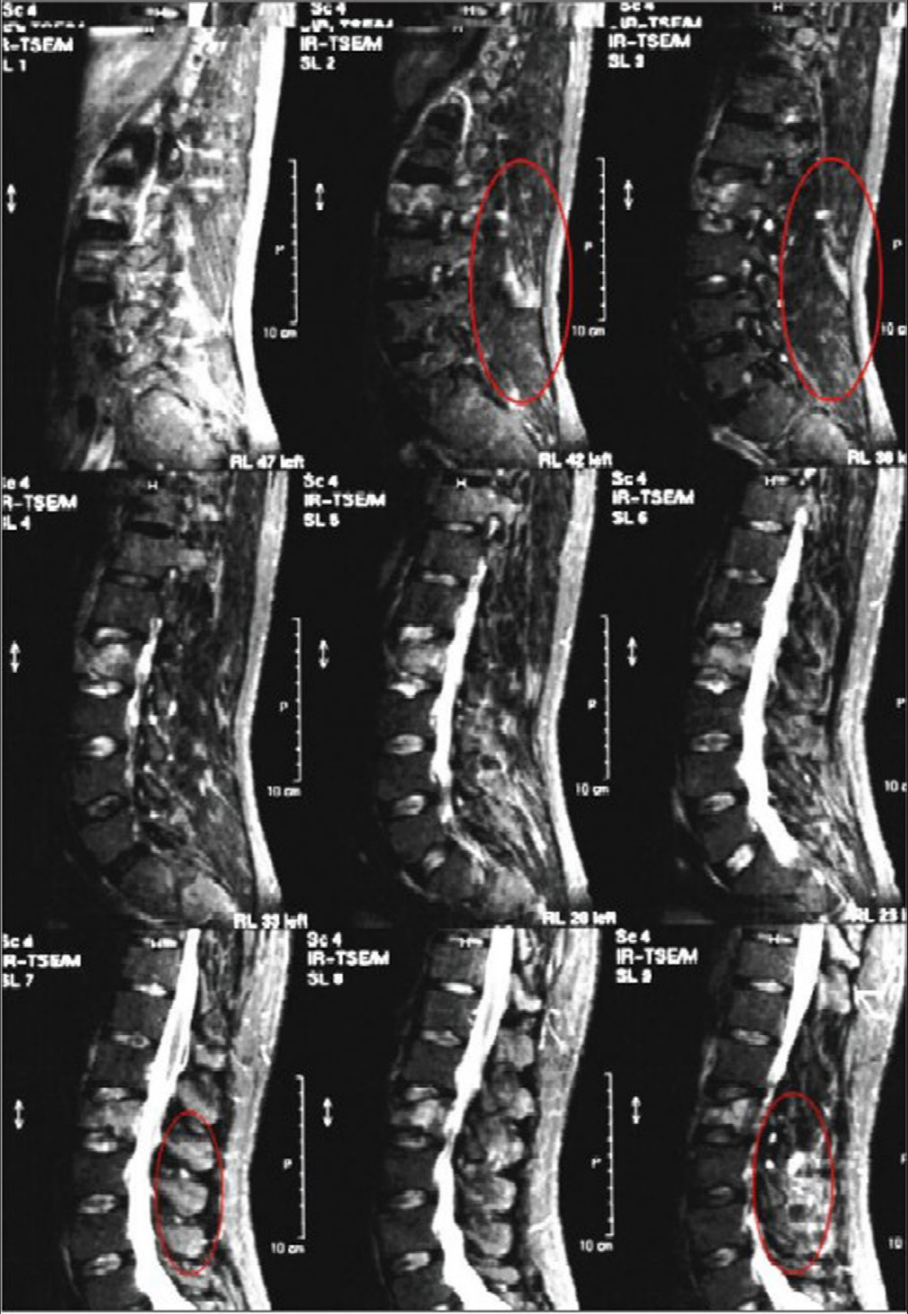
MRI-STIR (MRI-short tau inversion recovery) study shows hyper-intense signal in the interspinous ligaments suggestive of ligamentous damage

**Figure 1d:**
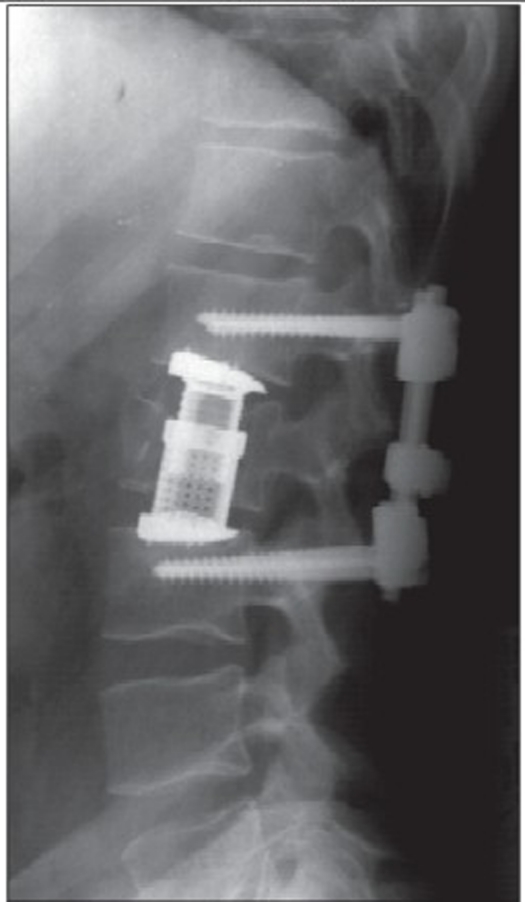
Postop X-ray shows the posterior stabilization and L1 vertebral body replacement
- group III (n = 20): “B or C” type fractures with LSC scoring less then 6 points, treated with only the posterior approach

When applied solely without any fusion or with monosegmental fusion in combined procedures, the internal pedicular fixator was removed after an average period of 15 months. Anterior implants were not removed. Patients in all groups were followed systematically with regards to subjective, clinical and radiographic results. Followup examination was performed six and 12 weeks and six and 12 months postoperatively and then yearly. Subjective assessment was based on self-evaluation of daily activities; objective followup parameters were assessed according to Prolo´s functional and economical scale. This study exclusively compares the early postoperative and the final radiographic results (endplate angle). In light of this objective, loss of correction (increase of kyphosis), possible implant failure and the fusion rate in patients with an anterior fusion were evaluated. Fusion assessment was based on analysis of lateral plain radiographs. Patients were followed for at least 22 months after the operation; the longest followup was 38 months.

## RESULTS

**Figure 2:**
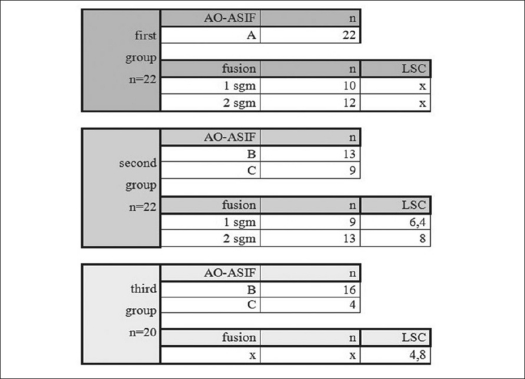
Survey of fracture types, fusion extent and LSC points in individual groups

**Figure 3:**
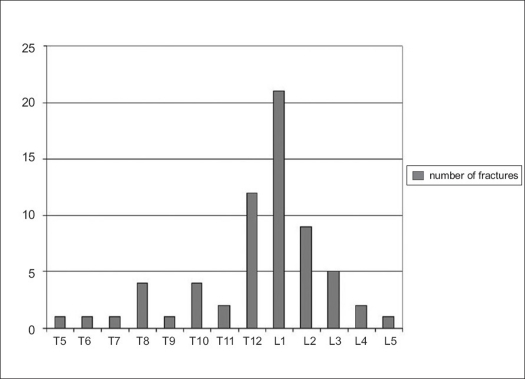
Bar diagram showing distribution of fracture levels

Type B fractures (29 cases) were the most frequent type of fractures. Survey of diagnoses, fusion extent and LSC points in all three groups are shown in Figure 2. Distribution of fractured vertebrae in the thoracolumbar spine has a typical layout [Figure 3]. Fusion rate in anterior procedures (group I) was 95%. Value of the same parameters in patients with combined postero-anterior procedures (group II) was 89% in patients with monosegemental fusion and 92% in those where a fusion was created bi-segmentally.

**Figure 4:**
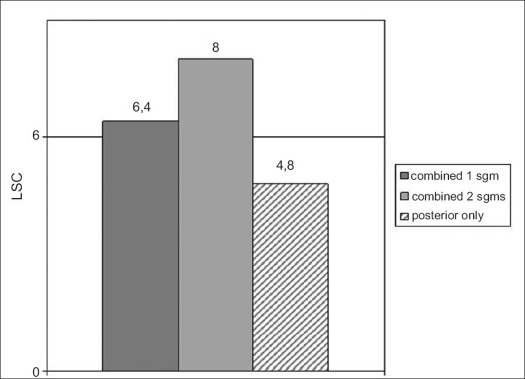
Bar diagram showing average LSC scores in combined mono- and bi-segmental fusions (group II) and in single posterior stabilizations (group III)

The average values of LSC scoring in fractures treated with combined procedures and with indication for mono- and bi-segmental fusion (group II) were 6.4 and 8.0 respectively. The average LSC score in fractures treated with only posterior stabilization (group III) was 4.8 [Figure 4]. Neither instrumentation failure nor any significant loss of reduction was observed in the first and second groups. In the third group, the mean loss of reduction (increase of kyphosis) was 3.1°.

## DISCUSSION

The technology of spine fracture imaging has significantly improved in recent years. Nevertheless, high-quality X-rays are still essential in the projection of basic shape parameters of the spine and the fracture and are the most accurate tool for evaluation of treatment results. Computer tomography (CT) perfectly depicts the whole vertebra and is an important guide for surgical planning. It gives information about bone fragments within the spinal canal. So far, there is no other imaging technology that is more useful than CT to examine facet relationships. Sagittal reconstruction makes it possible to evaluate the angle of kyphosis and the shape of the spinal canal narrowing. Magnetic resonance imaging (MRI) is dominant in soft tissue imaging: intervertebral discs (herniation, laceration), vessels (thrombosis), nerve roots (compression, interruption) and mainly the spinal cord (edema, ischemia, myelomalacia, hemorrhage, compression). This is a very important, noninvasive, diagnostic method for the evaluation of ligamentous injury. The AO-ASIF classification system is not applicable in preoperative decision-making without an MRI examination.^11^

The effort to understand the principles of spine stability led to the theory of three columns initially proposed by Denis for thoracolumbar fractures,^13^ but applicable for the whole spine^14^ and subsequently produced a number of classification systems.^15^ A very illustrative concept was published by Magerl.^16^ In recent years, two classification systems are predominantly used.

**Figure 5:**
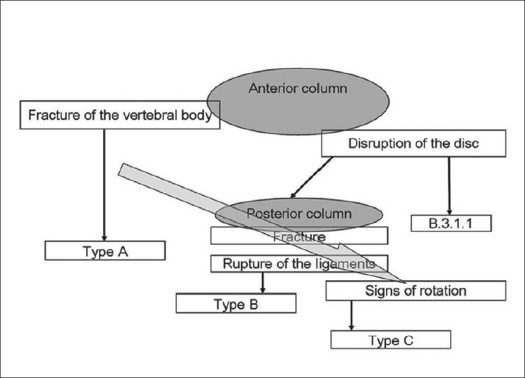
Algorithm for classifying thoracolumbar injuries according to the AO-ASIF classification^14^

The “comprehensive classification of thoracolumbar fractures”, also known as the AO-ASIF classification of spine fractures is based on the two column theory and divides all fractures into three categories, which are further sub divided into 55 groups, to define the various fractures of the thoracic and lumbar spine.^12^ The important feature of this classification is the information about the type of instability encountered. A simple algorithm is used to assess this parameter as shown in Figure 5 where a bold arrow shows the direction from stable to unstable fractures, *e.g.,* a decrease in residual fracture stability.

The reliability of the AO-ASIF classification was tested by Blauth *et al*.^17^ The reliability rate was 67% (41-97%) when basic categories (A, B, C) were used. High reliability was found during the evaluation of A category injuries. The study revealed a satisfying correlation in the determination of the method of treatment, pointing out at the same time, that indication of surgical approach (anterior, posterior and combined) is currently the most important goal. Nevertheless, the AO-ASIF classification cannot be accurately used preoperatively unless an MRI is performed. Leferink *et al*. reported the finding that 30% of type B fractures (AO-ASIF classification) are initially overlooked due to reliance on the AO-ASIF classification alone.^11^

**Figure 6:**
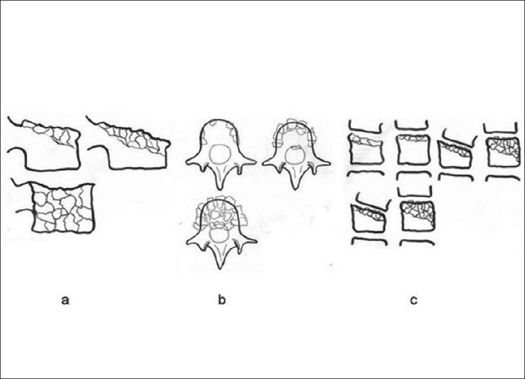
The Load Sharing Classification: a) the amount of damaged vertebral body, b) the spread of the fragments in the fracture site, c) the amount of corrected traumatic kyphosis.^9^

The second classification system is the “Load Sharing Classification” (LSC) devised by McCormack, Karaikovic and Gaines,^9^ whose aim is to set a guideline for indicating the method of treatment. This classification involves only injuries to the anterior spinal column. The LSC was devised based on data from a group of 28 patients with three-column fractures who were operated during the period of 1986-1988 with pedicular screw insertion and with 3-4 years of followup after surgery. There was a subgroup of ten patients within these 28 patients, who had pedicular screw breakage. Retrospective analysis of X-ray images, CT axial and sagittal reconstruction scans discovered that the risk of breakage correlated with significant damage of the vertebral body. Subsequently, the authors proposed a scoring system that considers a) the amount of the damaged vertebral body, b) the spread of the fragments in the fracture site and c) the amount of corrected traumatic kyphosis [Figure 6]. Each of these three parameters is rated from 1 to 3 points. Accumulation of more than 7 points becomes an indication for anterior fusion. However, this classification also covers fractures of the “pincer type” and “coronal split” that may be rated with 6 and more points. This means that in some cases the indication for anterior fusion comes with a lower LSC scoring.

Preoperative analysis of bony fracture anatomy with LSC is useful in determining candidates for short segment posterior instrumentation, short segment anterior stabilization or short segment posterior stabilization and anterior fusion with strut graft. The classification does not grade ligament damage and is not related to the mechanism of injury. It is a helpful adjunctive tool that can complement but not replace other forms of classification.^18^

The surprisingly high proportion of B-type fractures [Figure 2] observed is most probably due to the high sensitivity of MRI. It is obviously desirable to refine the use of this diagnostic method to detect real unstable injuries reliably among many other pathological findings. Nevertheless, the high sensitivity guaranteed the safety of the fracture management. Data from literature shows that isolated posterior stabilization is associated with a higher risk of loss of correction and instrumentation failure.^6^–^9^ Low grade of loss of correction in our group III correlates well with a low LSC score. Fusion in this study was assessed with plain lateral X-rays. Certainly, it is difficult to interpret radiographs of spine fusion and anterior interbody fusion. Plain radiographs are not ideal for analysis of spinal fusion, but until new and better diagnostic methods are available for clinical use, radiographs will remain the gold standard.^19^

The concept of surgical approach selection described in this paper is limited in several aspects. We do not use it in cases where the posterior approach is obviously the method of choice (osteoporosis, multiple vertebral fractures and urgent surgeries executed for acute spinal cord injury). It is also necessary to keep in mind the specific features of anterior approaches in the upper third of thoracic spine. Additionally, the anterior approach has more contraindications with respect to the patient's general condition.

## CONCLUSION

Complex fracture classification comprising a combination of the AO-ASIF and LSC classification methods helps to choose the surgical approach. A classification-related approach facilitates the prevention of treatment failure.
